# Severe mental illnesses and mortality following COVID-19 infection: Data linkage study using the Clinical Practice Research Database (CPRD).

**DOI:** 10.23889/ijpds.v7i3.2069

**Published:** 2022-08-25

**Authors:** Jayati Das-Munshi, Alex Dregan, Robert Stewart, Matthew Hotopf, Ioannis Bakolis, Laia Becares, Josephine Ocloo, Ruth Stuart, Elisa Impara

**Affiliations:** 1 IoPPN, KCL; 2 University of Sussex

## Background

The association of COVID-19 infection with death in people with severe mental illnesses (SMI), and the relationship to multimorbidities/ underlying health conditions ethnicity is unclear. Health records linked to COVID-19 tests data could help to inform this knowledge gap.

## Objective

To determine the risk of death in people with SMI following COVID-19 infection compared to reference groups and assess whether excess mortality is accounted through underlying health conditions or further elevated in minority ethnic groups.

## Design, setting and participants

Nationally representative cohort study using primary care data from the Clinical Practice Research Database (CPRD), with participants followed from the start of the pandemic in 2020, for 1.5 years, covering England, Wales and Northern Ireland. For consenting practices, CPRD data was linked to COVID-19 data Public Health England (PHE) Second Generation Surveillance System (SGSS), PHE COVID-19 Hospitalisation in England Surveillance System (CHESS), and Intensive Care National Audit and Research Centre (ICNARC) data on COVID-10 intensive care admissions. The cohort comprised 795,836 individuals, with 7,493 individuals with SMI and a positive COVID-19 test (“SMI/COVID-19”). Comparison groups were: 2,325 individuals with SMI/ testing negative for COVID-19 (“SMI/ non COVID-19”), 657,414 individuals from a non-SMI group/ testing positive for COVID-19 (“non-SMI/ COVID-19”), and 128,604 individuals from a non-SMI group/ testing negative for COVID-19 (“non-SMI/ non-COVID-19”).

## Exposures

SMI defined as the presence of schizophrenia, schizoaffective disorder, bipolar disorder, or affective disorders with psychosis, according to the International Classification of Mental Disorders (ICD-10). COVID-19 diagnoses identified through confirmed laboratory tests and clinical diagnoses.

## Outcomes

All-cause mortality

## Results

A higher proportion of SMI patients with COVID-19 were obese (37% versus 22% in the non-SMI/non-COVID-19 group), current smokers (27% versus 23% in the non-SMI/non-COVID-19 group), had underlying health conditions, and were Black Caribbean/ Black African (5% versus 1% in the non-SMI/non-COVID-19 group). Relative to the non-SMI/ non-COVID-19 group, the SMI/ COVID-19 group had an elevated risk of death (age and sex-adjusted hazard ratio (aHR) 5.03 (95%CI: 4.61-5.54)). This was elevated to a lesser extent, in the SMI/ non COVID-19 group (aHR: 1.93 (95%CI: 1.54-2.41)) and in the non-SMI/ COVID-19 group (aHR: 2.85 (95%CI: 2.72-2.98). Excess risk persisted after adjusting for tobacco use, weight and comorbidities. Mortality trends were similar across groups by ethnicity. Risk of death was highest for the SMI/ COVID-19 group during the first wave of infection in the UK, however excess mortality was still evident and substantially elevated at the second wave also.

## Conclusion/Implications

People living with SMI are at an increased risk of death compared to population controls; this excess risk is further elevated following COVID-19 infection, with similar trends by ethnicity. Underlying health conditions only partially account for deaths following COVID-19 infection in this group.

